# Continuous monitoring of the bronchial epithelial lining fluid by microdialysis

**DOI:** 10.1186/1465-9921-8-78

**Published:** 2007-11-01

**Authors:** Stig S Tyvold, Erik Solligård, Oddveig Lyng, Sigurd L Steinshamn, Sigurd Gunnes, Petter Aadahl

**Affiliations:** 1 Department of Anesthesia and Intensive Care, St. Olavs Hospital, Trondheim, Norway; 2 Department of Circulation and Medical Imaging, Norwegian University of Science and Technology, Trondheim, Norway; 3 Department of Cancer Research and Molecular Medicine, Norwegian University of Science and Technology, Trondheim, Norway; 4 Department of Lung Medicine, St. Olavs Hospital, Trondheim, Norway; 5 Department of Heart and Lung Surgery, St. Olavs Hospital, Trondheim, Norway

## Abstract

**Background:**

Contents of the epithelial lining fluid (ELF) of the bronchi are of central interest in lung diseases, acute lung injury and pharmacology. The most commonly used technique broncheoalveolar lavage is invasive and may cause lung injury. Microdialysis (MD) is a method for continuous sampling of extracellular molecules in the immediate surroundings of the catheter. Urea is used as an endogenous marker of dilution in samples collected from the ELF. The aim of this study was to evaluate bronchial MD as a continuous monitor of the ELF.

**Methods:**

Microdialysis catheters were introduced into the right main stem bronchus and into the right subclavian artery of five anesthetized and normoventilated pigs. The flowrate was 2 μl/min and the sampling interval was 60 minutes. Lactate and fluorescein-isothiocyanate-dextran 4 kDa (FD-4) infusions were performed to obtain two levels of steady-state concentrations in blood. Accuracy was defined as [bronchial-MD] divided by [arterial-MD] in percent. Data presented as mean ± 95 percent confidence interval.

**Results:**

The accuracy of bronchial MD was calculated with and without correction by the arteriobronchial urea gradient. The arteriobronchial lactate gradient was 1.2 ± 0.1 and FD-4 gradient was 4.0 ± 1.2. Accuracy of bronchial MD with a continuous lactate infusion was mean 25.5% (range 5.7–59.6%) with a coefficient of variation (CV) of 62.6%. With correction by the arteriobronchial urea gradient accuracy was mean 79.0% (57.3–108.1%) with a CV of 17.0%.

**Conclusion:**

Urea as a marker of catheter functioning enhances bronchial MD and makes it useful for monitoring substantial changes in the composition of the ELF.

## Background

The epithelial lining fluid of the lung is important in the understanding mechanisms in acute lung injury, inflammatory lung diseases, cardiac failure, and in pharmacokinetic studies.

Today there are no established methods of direct continuous monitoring of the epithelial lining fluid of the lower respiratory tract. The epithelial lining fluid of the bronchi has been examined by bronchioalveolar lavage (BAL), direct aspiration, microsampling, and exhaled breath condensates. BAL and other bronchoscopic techniques have in common that they are invasive, may create lung injury, are based on single or intermittent samples and therefore have limited value as continuous and dynamic monitors of the epithelial lining fluid of the lung. The exhaled breath condensates technique is non-invasive and continuous, but is indirect and best suited for collection of non-volatile hydrophilic solutes[1].

Microdialysis is a method for continuous sampling of extracellular molecules in the immediate surroundings of the catheter. The technique is based on the principle of diffusion of substances along a concentration gradient through a semipermeable membrane on a thin catheter with an outer diameter of 0.6 mm. Microdialysis has routinely been used in parenchymatous organs and tissues, but has also gained widespread acceptance in hollow organs. Intestinal endoluminal microdialysis has been used as a continuous monitor of intestinal dysfunction both in experimental [2-4] and in clinical settings[5]. As far as we know there is only one published article on bronchial microdialysis in rats for pharmacokinetic measurements of aminoglycosides[6]. The value of microdialysis as a continuous and dynamic monitor of the time to time changes in the epithelial lining fluid has not previously been evaluated.

The recovered amount of epithelial lining fluid by various techniques, especially BAL, has been estimated by endogenous and exogenous markers of dilution. Urea (60 Da) is a small molecule in equilibrium in all body compartments. The recovered concentration of urea has been used as an indicator of the recovered concentration of epithelial lining fluid in the dialysate [7-10]. The arteriobronchial urea gradient has been used to calculate the absolute concentration of the molecules measured in the epithelial lining fluid by BAL and the ureagradient has also been used to calculate the absolute concentration of molecules measured in the extracellular fluid[9,11].

Lactate (90 Da) is a small molecule which has previously been studied as a marker of intestinal barrier dysfunction[2]. In the intestines lactate only passes the blood-luminal barrier of the intestines when the intestines suffer an ischemia-reperfusion injury[4]. The question is if lactate freely passes the blood-bronchial barrier.

Molecular transport across rat epithelial monolayer by the paracellular route has previously been investigated with the macromolecule fluorescein isothiocyanate dextran 4000 Da (FD-4)[12,13]. According to the present literature an increased leakage of FD-4 is believed to be via the paracellular route by changes in the composition and confirmation of the tight junctions of the epithelium and the endothelium in response to stimuli[14,15]. In this study, we used FD-4 to see if larger molecular size influences the gradient across the paracellular blood-bronchial barrier.

The aim of our study was to evaluate bronchial microdialysis as a possible method for continuous and dynamic monitoring of the time to time changes in the composition of the epithelial lining fluid. Lactate and FD-4 were measured during two levels of steady-state concentrations in anesthetized pigs under positive pressure ventilation. Arteriobronchial urea gradient was used as a correction factor to calculate the absolute concentration of lactate and FD-4 in the bronchial epithelial lining fluid.

## Methods

### Anesthesia and surgical preparation

Five outbred pigs (Norwegian Landrace 50%, Duroc 25%, Yorkshire 25%) (range 22–31 kg) were acclimatized and treated in accordance with the "European Convention for the Protection of Vertebrate Animals used for Experimental and Other Scientific Purposes", Strasbourg, 18.III.1986. The pigs fasted overnight with free access to water prior to the experiment. They were premedicated with intramuscular diazepam 10 mg (Stesolid, Dumex-Alpharma, Copenhagen, Denmark) and azaperon 400 mg (Stresnil, Janssen-Cilag, Vienna, Austria), and anesthesia was induced with atropine 1 mg (Nycomed Pharma AS, Oslo, Norway), ketamin HCl 10 mg/kg (Parke-Davis, Solna, Sweden) and thiopenthal sodium 10 mg/kg (Pentothal, Abbot Scandiavia AB, Solna, Sweden).

Tracheostomy was performed with the pig in the supine position, and an endotracheal tube 8.0 was inserted 5 cm.

The pigs received total intravenous anesthesia with a continuous infusion of fentanyl 20 to 30 μg/kg/hour (Fentanyl, Pharmalink, Spanga, Sweden) and ketamin HCl 8 to 12 mg/kg/hour. Pancuronbromide 2 mg/ml (Pavulon, Organon Teknika, Baxtel, The Netherlands) was used as needed to eliminate spontaneous ventilation.

The pigs were ventilated in pressure control mode with a positive end expiratory pressure of 7 cmH2O, a peak inspiratory pressure less than or equal to 20 cmH2O and a tidal volume of 6 to 8 ml/kg (Servo Ventilator 900C, Siemens-Elema, Sweden)[16,17].

After a recruitment maneuver of 3 breaths at 40 cmH2O, respiratory rate was adjusted to an arterial partial pressure of carbon dioxide (PaCO_2_) of 4.5 to 5.5 kPa in baseline. Fraction of inspired oxygen (FiO_2) _was maintained at 25% during the experiment. Similar recruitment maneuvers were performed when the bronchial microdialysis catheter was in position. In 2 out of 5 pigs a recruitment maneuver was necessary during the experimental protocol due to spontaneous ventilation in conflict with the ventilator and derecruitment. This was solved with pancuronium bromide, increased infusion of the anesthetetic agents and a recruitment maneuver to reestablish the lung compliance as measured by lung tidal volume/peak inspiratory measure.

The pigs received an intravenous infusion of heated (37°C) Ringers acetate at 14 to 18 ml/kg/hour throughout the experiment. Rectal temperature was maintained within the normal range (37 to 39.6°C) by a heating mattress[18].

The right femoral artery was cannulated with a triple lumen catheter (Certofix-Trio S715, Braun, Melsungen, Germany) for sampling of arterial blood gases and invasive blood pressure monitoring (Tram-rac 4A/Tram-scope 12C, Marquette Electronics, USA). A single lumen catheter (Sekalon-T 16G, Becton Dickinson, Singapore) was inserted in the femoral vein for blood sampling. The right carotid artery and the right jugular vein were surgically prepared. A pulmonary artery catheter (Swan Ganz CCOmbo 7.5 Fr, Edwards Lifescience, USA) was inserted, and positioned using the pulmonary arterial blood pressure curve and the pulmonary artery wedge pressure curve. Continuous cardiac output and central venous saturation were monitored (Vigilance, Edwards Lifescience, USA).

### Microdialysis

Alongside the 8.0 endotracheal tube a 4.0 endotracheal tube was inserted into the trachea and advanced to a position 1 cm proximal to the carina confirmed by fiberoptic bronchoscopy. A thread was used to secure the two tracheal tubes and to avoid air leakage. The microdialysis catheter (CMA Custom made, 10 mm membrane length, 100 kDa cut-off, outer diameter 0.6 mm, CMA Microdialysis, Stockholm, Sweden) was introduced through the 4.0 endotracheal tube and guided fiberoptically into the left main stem bronchus. The microdialysis catheter was gently guided forward until wedged and then retracted 0.5 to 1 cm to avoid atelectasis. The proximal opening of the 4.0 endotracheal tube was sealed with wax to avoid air leakage.

Another microdialysis catheter (CMA Custom made, 10 mm membrane length, 100 kDa cut-off, outer diameter 0.6 mm, CMA Microdialysis, Stockholm, Sweden) was inserted into the right subclavian artery through a venous catheter (Optiva2 18G, Medex, Great Britain) in the right brachial artery.

Both microdialysis catheters were connected to microdialysis pumps (CMA 107, CMA Microdialysis AB, Stockholm, Sweden) and perfused by sterile phosphate buffered saline (PBS) at a flow rate of 2 μl/min.

The microdialysis catheters were perfused with a flow of 2 μl/min. The volume on the inside of the membranous part of the microdialysis catheters used in this study was less than 1.4 μl. Thus the time period of membrane contact of the microdialysis perfusion fluid was short.

The catheters were perfused in situ for ≥60 minutes before the experiment was started. Samples from each microdialysis catheter were collected in microvials that were exchanged every 60 minutes, and analyzed online for lactate and urea on the CMA 600 (CMA Microdialysis AB, Stockholm, Sweden), using the peroxidase methodology.

**Figure 1:**
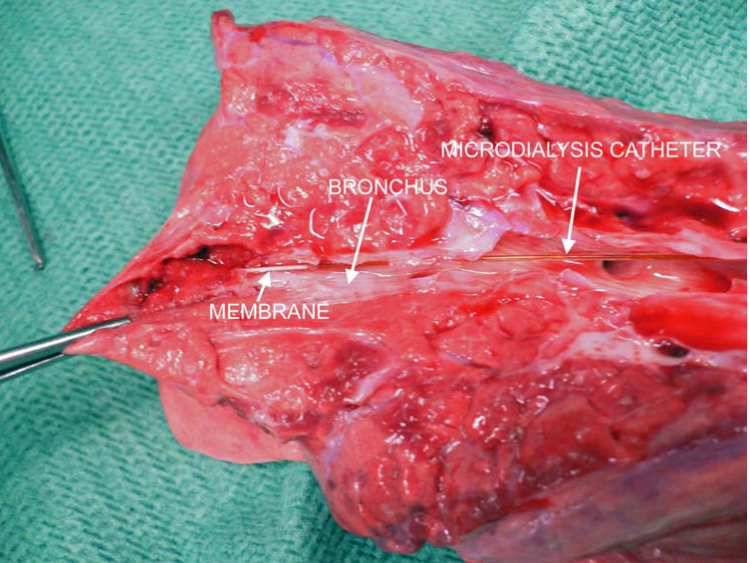
**The microdialysis catheter in situ**. The picture shows the microdialysis catheter in the distal bronchus. The distal white part is the microdialysis membrane. It is this part of the catheter that is in contact with the epithelial lining fluid and collects molecules by diffusion.

To verify the position of the catheter at the end of the experiment, the microdialysis catheter either was perfused with methylene blue and the bronchial tree dissected or the catheter was left in situ and the bronchial tree dissected (figure 1).

### Experimental protocol

After surgical preparations the animals were allowed to stabilize for 60 minutes.

### Lactate

**Figure 2:**
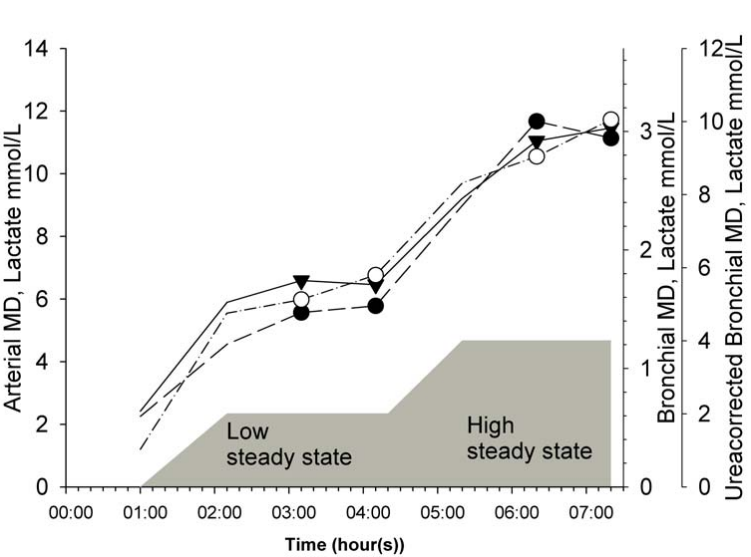
**Overview of the lactate infusion with two steady states**. Data are presented as mean lactate values by arterial microdialysis (solid line) with mean values in steady state (filled triangles), bronchial microdialysis (long dash line) with mean values in steady state (filled circles) and ureacorrected bronchial microdialysis (dash-dot line) with mean values in steady state (open circles). The circles and the triangles represent the time where the microdialysis vials were exchanged for the steady state samples. The gray area is a graphical presentation of the intravenous lactate infusion. During the low steady state the infusion of sodium lactate was gradually increased to maintain an arterial blood lactate of ~5 mmol/L. During the high steady state the infusion of sodium lactate was increased to maintain an arterial blood lactate of ~10 mmol/L.

An infusion of sodium lactate ~50% (Merck KGaA, Darmstadt, Germany) was adjusted to an arterial blood lactate concentration of ~5 mmol/l for 120 minutes. Thereafter, the infusion rate was increased to an arterial blood lactate concentration of ~10 mmol/l (figure 2). Arterial blood was collected every 15 minutes throughout the experiment to establish a steady-state of lactate and PaCO_2 _(ABL700, Radiometer Copenhagen, Denmark).

### Fluorescein isothiocyanate dextran 4000 Da infusion

**Figure 3:**
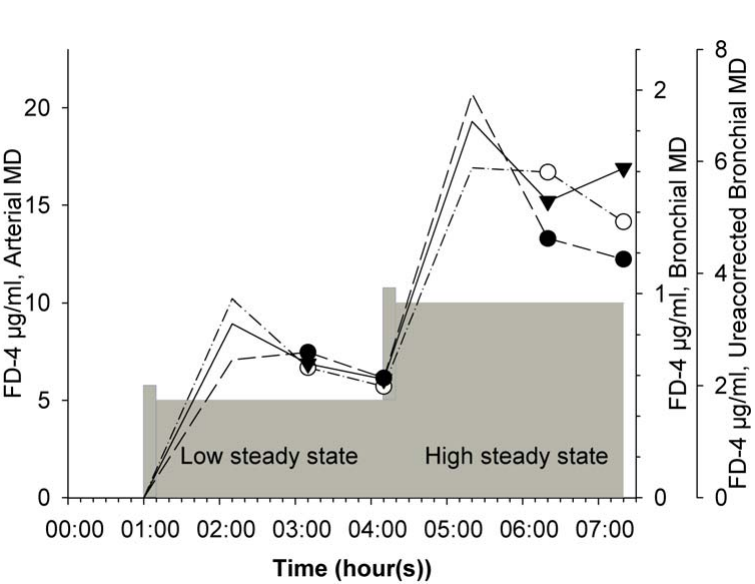
**Overview of the fluorescein isothiocyanate dextran 4000 Da (FD-4) infusion with two steady states**. Data are presented as mean FD-4 values by arterial microdialysis (solid line) with mean values in steady state (filled triangles), bronchial microdialysis (long dash line) with mean values in steady state (filled circles) and ureacorrected bronchial microdialysis (dash-dot line) with mean values in steady state (open circles). The circles and the triangles represent the time where the microdialysis vials were exchanged for the steady state samples. The gray area is a graphical presentation of the intravenous FD-4 infusion. The infusion was started with an intravenous bolus of 10 μg/kg over 10 minutes. In the low steady state an infusion of FD-4 at 5 μg/kg/hour was maintained. After the low steady state a new intravenous FD-4 bolus of 10 μg/kg over 10 minutes was infused. In the high steady state an infusion of FD-4 at 10 μg/kg/hour was maintained.

A solution of FD-4 (Sigma Chemical, St. Louis, MO, USA) in PBS was prepared fresh each day at a concentration of 10 mg/ml. The FD-4 infusion started with an intravenous loading dose of 10 mg/kg over 10 min followed by an infusion of 5 mg/kg/hour for 180 minutes. Then parallel to the FD-4 infusion at 5 mg/kg/hour a new bolus of 10 mg/kg was given. After the bolus the infusion was increased to 10 mg/kg/hour for the last 180 minutes of the experiment (figure 3).

Beginning during in the stabilizing period, 0.5 ml of arterial blood was sampled every 60 minutes throughout the experiment. Fluorescence of plasma and microdialysate samples was measured on a fluorescence spectrophotometer (Fluoroskan II, Labsystem).

### Arteriobronchial urea gradient

Urea concentrations are the same throughout various body compartments. The arteriobronchial urea gradient in each sampling period is a measure of the microdialysis catheter functioning in the bronchial as opposed to the arterial compartment.

### In vivo recovery

Blood lactate was measured every 30 minutes. Mean arterial blood concentration of lactate during a 60-minutes period was defined as the mean of the value at 0, 30 and 60 minutes for the period.

Arterial blood FD-4 was measured every 60 minutes. Mean arterial blood concentration of FD-4 during a 60-minutes period was defined as the mean of the value at 0 and 60 minutes for the period

Arterial microdialysis was sampled over the same 60-minute period.

In vivo recovery was calculated as [Molecule]_arterial microdialysis_/[Molecule]_arterial blood_.

### In vitro microdialysis

Lactate wells were prepared in PBS at lactate concentrations of ~1, ~5 and ~10 mmol/L. Three microdialysis catheters (CMA20, 10 mm membrane length, 100 kDa cut-off, outer diameter 6 mm, CMA microdialysis, Stockholm, Sweden) were perfused with PBS at 2 μl/min and put in the first well, lactate ~1 mmol/L, for 60 minutes to stabilize. Thereafter vials were exchanged every 60 minutes for 180 minutes. Samples from the wells were collected and analyzed at the beginning and at the end of the experiment. The procedure was then repeated for lactate ~5 and ~10 mmol/L. All lactate samples were analyzed on the CMA 600 (CMA Microdialysis AB, Stockholm, Sweden).

The procedure was repeated for FD-4 wells prepared with PBS at FD-4 concentrations of ~10, ~50 and ~150 μg/ml. This setup gave good and stable volume recovery with FD-4 samples being analyzed in triplicate or quadruplicate, each well containing 25 μl (Fluoroskan II, Labsystem).

In vitro recovery was calculated as [Molecule]_well microdialysis_/[Molecule]_well_.

### Statistics and calculations

Arterial microdialysis values were considered to be the real arterial concentration during each sample period. Bronchial values are presented with and without correction by the arteriobronchial urea gradient. Ureacorrection was done in accordance to the formula in Rennard's article[9]. ([Urea]_arterial_/[Urea]_bronchial_)/([Molecule]_arterial_/[Molecule]_bronchial_).

Evaluation of bronchial microdialysis and urea-corrected bronchial microdialysis as an approximation of arterial microdialysis are presented as accuracy. The bronchial microdialysis values, corrected and uncorrected, are presented as fractions of the arterial microdialysis values. Accuracy = [Molecule]_bronchial_/[Molecule]_arterial_. To evaluate the benefit of the urea correction, the coefficient of variation was used. Coefficient of variation = Standard deviation/Mean·100.

Calibrations of the microdialysis catheters were done by in vitro experiments and in vivo calculations. In vitro relative recovery = [Molecule]_well microdialysis_/[Molecule]_well_. In vivo relative recovery = [Molecule]_arterial microdialysis_/[Molecule]_arterial blood_.

The data are presented as values and mean ± 95% confidence interval. Paired t-test was used to compare means between arterial, bronchial and urea-corrected bronchial microdialysis with significance defined as p < 0.05.

## Results

### Circulatory and respiratory measurements

Heart rate, mean artery pressure, cardiac output, central venous oxygen saturation and partial pressure of carbondioxide remained stable throughout the entire experiment (table 1).

**Table 1:** Central hemodynamic and respiratory variables.

Parameter	Stabilizing	Low steady state	High steady state
Mean arterial pressure (mmHg)	65 ± 3	64 ± 2	60 ± 1
Heart rate (beats/min)	81 ± 15	88 ± 6	89 ± 3
Cardiac output (l/min)	4.2 ± 0,9	4.8 ± 0.4	4.9 ± 0.4
Central venous oxygen saturation (percent)	59 ± 5	62 ± 3	60 ± 3
Arterial partial pressure of carbon dioxide (kPa)	4.81 ± 0.23	4.78 ± 0.09	4.94 ± 0.11

Values are mean ± 95% confidence interval.

### Recovery measurements

The in vivo lactate relative recovery was 105.9 ± 7.2%. The in vivo FD-4 relative recovery was 28.8 ± 4.4%.

The in vitro lactate relative recovery was 54.9 ± 3.2%. The in vitro FD-4 relative recovery was 14.2 ± 1.0%.

### Arteriobronchial urea gradient

Arterial urea by microdialysis was 3.69 ± 0.59 mmol/L. Bronchial urea by microdialysis was 1.19 ± 0.21 mmol/L. Arteriobronchial urea gradient was 3.78 ± 0.85.

### Lactate

**Figure 4:**
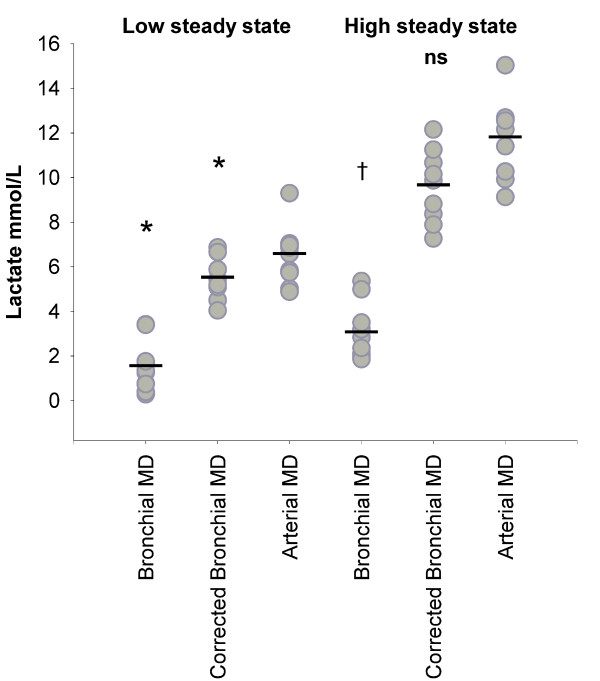
**Microdialysate lactate**. All values (gray circles) and mean (-) are presented. Lactate values by corrected bronchial microdialysis are corrected by the arteriobronchial urea gradient. * Paired T-test showed significant difference from arterial microdialysis at low steady state (arterial blood lactate ~5 mmol/L) (p < 0.05). † Paired t-test showed significant difference from arterial microdialysis at high steady state (arterial blood lactate ~10 mmol/L) (p < 0.05). ns Non significant from arterial microdialysis at high steady state (arterial blood lactate ~10 mmol/L).

During the steady-state periods lactate measured by arterial microdialysis was 6.5 ± 0.9 and 11.8 ± 1.4 mmol/L, by bronchial microdialysis 1.5 ± 0.8 and 3.0 ± 0.9 mmol/L and by urea-corrected bronchial microdialysis 5.5 ± 0.8 and 9.6 ± 1.3 mmol/L respectively. There was an arteriobronchial gradient of 1.2 ± 0.1 for lactate as measured by arterial microdialysis and bronchial microdialysis, corrected by the arteriobronchial urea gradient. Steady-state values and mean values for arterial lactate, bronchial lactate and urea-corrected bronchial lactate, as measured by microdialysis are presented in figure 4. Paired t-test show a significant difference (p < 0.05) between lactate by arterial microdialysis and urea-corrected bronchial microdialysis at low steady state (blood lactate ~5 mmol/L).

**Figure 5:**
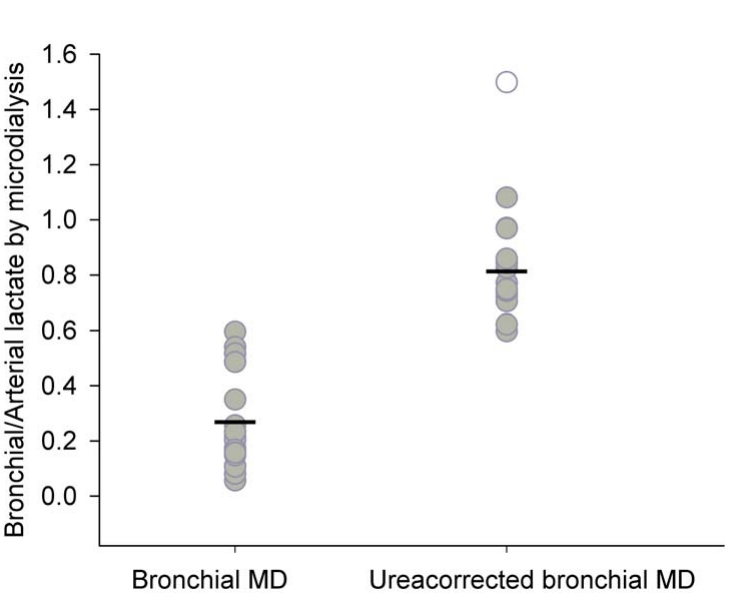
**Accuracy of bronchial microdialysis of lactate**. All values (gray circles) and mean (-) are presented. One value (open circle) is excluded as an outlier due to extreme deviation in the arterial microdialysis value. The accuracy of bronchial microdialysis with a continuous lactate infusion was mean 0.26 ± 0.08 with a coefficient of variation of 62.6%. The accuracy of bronchial microdialysis with a continuous lactate infusion and a correction by the arteriobronchial urea gradient was mean 0.81 ± 0.06 with a coefficient of variation of 17.0%. The reduced coefficient of variation after correction by the arteriobronchial ureagradient sustains the ureacorrection as useful correction factor to estimate the absolute concentrations of molecules in the epithelial lining fluid as measured by microdialysis.

The accuracy of bronchial microdialysis with a continuous lactate infusion was mean 0.26 ± 0.08 with a coefficient of variation of 62.6%. The accuracy of bronchial microdialysis with a continuous lactate infusion and a correction by the arteriobronchial urea gradient was mean 0.81 ± 0.06 with a coefficient of variation of 17.0% (figure 5). The reduction in the coefficient of variation is in accordance with urea being a molecule in almost immediate equilibrium within all body compartments and thereby is a correction factor of bronchial microdialysis catheter functioning.

Non-microdialysis data are presented in table 2.

**Table 2:** Non-microdialysis data.

Parameter	Low steady state	High steady state
Lactate (mmol/L)	5.9 ± 0.4	11.2 ± 0.3
Fluorescein isothiocyanate dextran 4000 Da (μg/ml)	25.2 ± 3.8	49.5 ± 6.7

Lactate was measured in blood and fluorescein isothiocyanate dextran 4000 Da was measured in plasma. Non-microdialysis data are intermittent samples and not continuous measurements. Values are mean ± 95% confidence interval.

### Fluorescein isothiocyanate dextran 4000 Da

**Figure 6:**
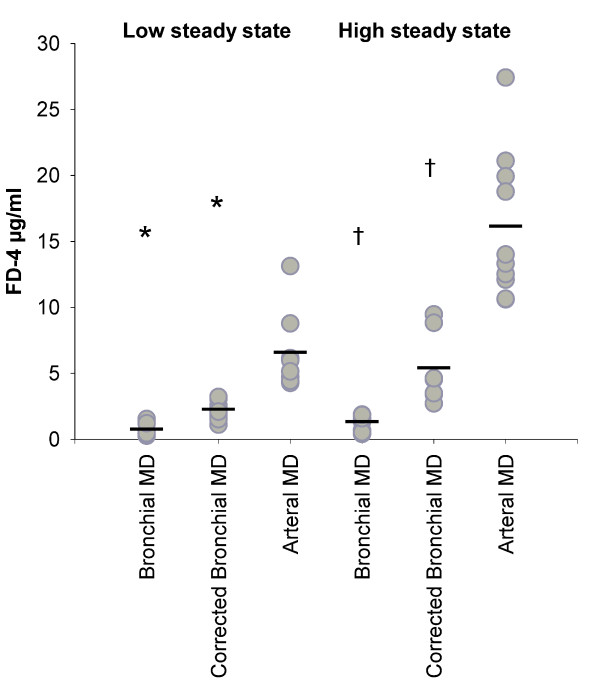
**Microdialysate fluorescein isothiocyanate dextran 4000 Da**. All values (gray circles) and mean (-) are presented. Fluorescein isothiocyanate dextran 4000 Da (FD-4) values by corrected bronchial microdialysis are corrected by the arteriobronchial urea gradient. * Paired t-test showed significant difference from arterial microdialysis at low steady state (FD-4 5 μg/kg/hour) (p < 0.05). † Paired t-test showed significant difference from arterial microdialysis at high steady state (FD-4 10 μg/kg/hour) (p < 0.05).

During the steady-state periods FD-4 measured by arterial microdialysis was 6.5 ± 1.9 and 16.0 ± 4.0 μg/ml, by bronchial microdialysis 0.7 ± 0.3 and 1.2 ± 0.5 μg/ml and by urea-corrected bronchial microdialysis 2.2 ± 0.6 and 5.3 ± 2.5 μg/ml respectively. There was an arteriobronchial gradient of 4.0 ± 1.2 for FD-4 as measured by arterial microdialysis and bronchial microdialysis, corrected by the arteriobronchial urea gradient. Steady-state values and means for arterial FD-4, bronchial FD-4 and ureacorrected bronchial FD-4, as measured by microdialysis, are presented in figure 6. There was a defined barrier between blood and bronchi for the diffusion of FD-4.

The accuracy of bronchial microdialysis with a continuous FD-4 infusion was mean 0.09 ± 0.03 with a coefficient of variation of 65.2%. The accuracy of bronchial microdialysis with a continuous FD-4 infusion and a correction by the arteriobronchial urea gradient was mean 0.35 ± 0.10 with a coefficient of variation of 54.8%. The reduction in the coefficient of variation was less than expected. Previous knowledge of FD-4 analyses on the Fluoroskan II is from experiments with wells containing 150 μl. Due to small volumes sampled by microdialysis, pilot in vitro studies were conducted with wells containing 15 μl, 25 μl and 40 μl. With wells containing 15 μl there was a great variance and by visual inspection we saw that this small volume did not cover the bottom of the well. Wells containing 25 μl and 40 μl gave similar results and variance (data not shown). During analysis of the last fluorescein isothiocyanate results from the in vivo experiment, we registered that wells with higher concentrations of FD-4 increased the registered result of neighboring wells with lower concentration of FD-4. Due to lack of material the analysis could not be repeated and controlled.

Non-microdialysis data are presented in table 2.

### Individual correlation

**Figure 7:**
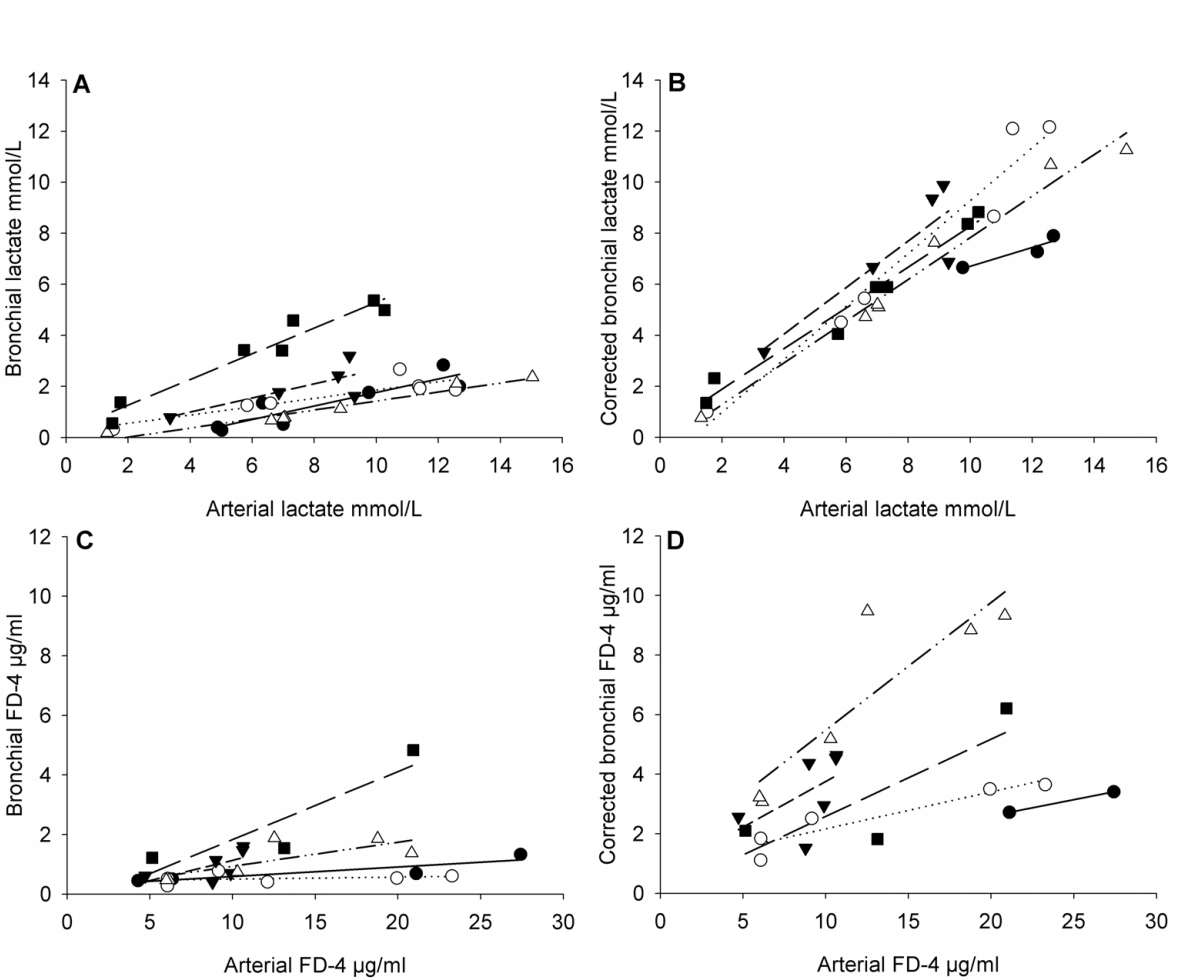
**XY-plot of bronchial and corrected bronchial microdialysis data against arterial microdialysis data of lactate and fluorescein isothiocyanate dextran 4000 Da**. All five pigs are represented; number one closed circles and line, number two open circles and dotted line, number three closed triangles and medium dashed line, number four open triangles and dash-dot-dot line, and number five closed squares and long dashed line. Plot A and B show arterial lactate along the X-axis and bronchial (R^2 ^0.82 ± 0.18) and ureacorrected bronchial lactate (R^2 ^0.91 ± 0.11) along the Y-axis respectively. Paired t-test of R^2 ^for the individual bronchial and ureacorrected bronchial lactate XY-plots showed significant difference (p < 0.05). Plot C and D show arterial fluorescein isothiocyanate dextran 4000 Da (FD-4) along the X-axis and bronchial (R^2 ^0.53 ± 0.38) and ureacorrected bronchial FD-4 (R^2 ^0.72 ± 0.34) along the Y-axis. Paired t-test of R^2 ^for the individual bronchial and ureacorrected bronchial FD-4 XY-plots was not significant.

To evaluate bronchial microdialysis as a tool of continuous monitoring all pigs are presented in XY-plots with individual values and correlation lines (figure 7). There was a significant improvement in the correlation between urea-corrected bronchial and arterial lactate values versus bronchial and arterial lactate values by paired t-test. For fluorescein isothiocyanate, the improvement was only a tendency and did not reach significance.

## Discussion

Extensive research on the bronchial epithelial lining fluid has been done to understand mechanisms of permeability, pharmacokinetics and pathophysiology. The lack of techniques for continuous monitoring and the possibility of causing lung injury have limited previous work to describe the dynamics of physiological phenomena. Lung microdialysis as previously described, with an open surgical technique and "interstitial" introduction of the microdialysis catheter by visual control, is only suitable for patients undergoing thoracic surgery [19-21].

This study indicates that bronchial microdialysis can be used as a continuous monitor of the epithelial lining fluid. Correction of bronchial microdialysis by the arteriobronchial urea gradient enhances bronchial microdialysis, as measured by lactate, by reducing the coefficient of variation. Correction by the arteriobronchial urea gradient makes bronchial microdialysis suitable as a monitor of substantial concentration changes in the epithelial lining fluid.

There is an ongoing controversy on how to estimate the amount of epithelial lining fluid recovered. Urea rapidly diffuses and equilibrates into the total volume sampled with BAL technique. Therefore recovered concentration of urea as a marker of recovered concentration of epithelial lining fluid has a tendency to overestimate the recovered concentration of epithelial lining fluid and hence underestimate the bronchial concentration of the measured molecule. Total BAL volumes in humans vary from 100 to 300 ml[9,22]. The tendency of overestimating the recovered volume of epithelial lining fluid also increases with increasing dwell time with BAL technique. The dwell time of BAL varies from about 3 to more than 10 minutes. Technetium-99 m diethylenetriaminepenta-acetic acid has been used as an alternative marker of recovered concentration of epithelial lining fluid without the problem of additional influx in the instilled lavage volume[23]. However the method is time consuming and this limits its applicability in both clinical and experimental settings. With small volume lavage (3 aliquots of 1 ml) with short dwell time (less than 1 minute) urea is a valid marker of dilution in BAL fluids in both the normal, diseased and recovering lung in infants, according to Dargaville et al[7].

With microdialysis, the additional volume to equilibrate was minimal (less than 1.4 μl), limited to the volume on the inside of the semi-permeable membrane of the catheter. With a perfusion flow of 2 μl/minute and the short time period of contact between the microdialysis perfusion fluid and the epithelial lining fluid, the arteriobronchial urea gradient was a reasonable correction factor for the recovered concentration of epithelial lining fluid. Thus it could be a correction factor to calculate the absolute concentrations of lactate and FD-4 in the epithelial lining fluid[7,10,11,23].

In vivo studies of transport of molecules are complicated by the metabolic activity of the organism. Molecules will to some extent be depositioned both extra- and intracelluarly. Metabolism in all tissues and renal clearance of the molecule one measures are factors that must be corrected and/or considered when the conclusions are drawn.

Lactate is depositioned in all body compartments, especially red blood cells and muscle[24]. The lung itself is a possible producer of lactic acid[25]. Urea (60 Da) and lactate (90 Da) differ in size and shape and may have different diffusion properties over the blood-bronchial barrier. Lactate passed the blood-bronchial barrier rather freely with a gradient of 1.2:1. Whether the gradient is a true blood-bronchial gradient is not known at present.

FD-4 crossed the blood-bronchial barrier with a gradient of about 4:1. As described previously in monolayer cell cultures of rat alveolar epithelial cells, FD-4 is transported through paracellular pores in human cultured alveolar epithelial cell monolayer[26]. FD-4 was chosen as a macromolecule with restricted transport through paracellular pores. Matsukawa showed this in rat alveolar epithelial cell monolayer[12]. As far as we know there is no previous study that describes FD-4 transport in vivo across the blood-bronchial barrier. In our study FD-4 had to cross intact endothelium, lung interstitium and lung epithelium. Due to methodological limitations with small volume samples, our FD-4 results varied. The blood-bronchial gradient was 4.0:1 ± 1.2. FD-4 was transported across the blood-bronchial barrier with restriction, in accordance with previous findings, but this requires further research to be confirmed.

The arteriobronchial urea gradient as a marker of bronchial catheter function reduced the coefficient of variation from 62.6% to 17.0% of the bronchial microdialysis as measured for lactate. The arteriobronchial urea gradient significantly improved correlation of arterial and bronchial lactate sampled over the same time interval in the same pig. A coefficient of variation of 17% still limits the use of bronchial microdialysis in the measurement of minor changes in lactate concentration of the bronchial epithelial fluid.

Bronchial microdialysis has some limitations. Bronchi are relatively stiff lumens with continuous movements during the respiratory cycle. The epithelial lining fluid is only a thin film. Microdialysis is developed for analyzing the composition of extracellular fluids in compact tissues with little movement. In the bronchi, one must assume that only varying parts of the microdialysis membrane are in contact with the epithelial lining fluid during the respiratory cycle. Thus, a marker of catheter functioning is necessary for characterizing each individual microdialysis catheter, measuring the recovered amount of epithelial lining fluid and the absolute concentrations of the molecules in the epithelial lining fluid.

With correction of bronchial microdialysis by the arteriobronchial urea gradient, the coefficient of variation of FD-4 decreased only slightly, from 65.2% to 54.8%. There was only a tendency toward improved correlation between arterial and bronchial FD-4 sampled over the same time interval in the same pig. The pilot studies on concentration and variation of different volumes on fluorescence spectrophotometry concluded that 25 μl in each well was sufficient to get precise numbers with a small degree of variation. But the FD-4 results from our study were not optimal with a high degree of variation. One of the problems was that wells with high concentration of FD-4 increased the measured FD-4 concentration in neighboring wells with low concentration of FD-4 according to our fluorescence spectrophotometer. This problem could be a contributing factor to the lack of precision with our method. The lack of reduction in the coefficient of variation of FD-4 by urea correction is probably due to methodological errors in analyzing FD-4 in small volumes by fluorescence spectrophotometry. Thus FD-4 measurement in small volumes still has to be refined to increase the precision of the method.

In vivo lactate recovery was more than 100% (105.9 ± 7.2%). The non-microdialysis samples were analyzed in whole blood. The microdialysis probe was placed in plasma in the subclavian artery. The plasma lactate is higher than the lactate value in erythrocytes[27].

In our laboratory we have shown that the CMA 600 (CMA Microdialysis AB, Stockholm, Sweden) and the ABL700 (Radiometer Copenhagen, Denmark) return different results from the same sample with a known lactate concentration (data not shown). The CMA600:ABL700 ratio for lactate in our laboratory is mean 1.13:1. The in vivo recovery percent for lactate must be evaluated with these factors in mind.

There was a major difference, about 2:1, between in vivo and in vitro recovery. In vitro the microdialysis catheter was placed in a well, collecting molecules from the immediate surroundings. In general, this means that there will always be a concentration gradient within the well around the membrane of the microdialysis catheter. Accordingly, there was a local concentration gradient and a smaller concentration of the measured molecule in the immediate surroundings of the microdialysis catheter. This effect increased with increasing perfusion volume per time unit. 2 μl/min is a relatively high perfusion rate with the microdialysis technique. In vivo, the catheter was in the arterial bloodflow with a continuous replacement of the fluid surrounding the microdialysis membrane so that the concentration of the measured molecule was the same in the immediate surroundings of the microdialysis membrane as in the rest of the fluid compartment.

Based on good volume recovery in the in vitro experiments, an isotonic crystalloid perfusion fluid was chosen. A microdialysis membrane with 100 kDa cut-off has large pores which easily can contribute to a loss of collected volume limiting the possibility for analyzing triplicates. The literature indicates that use of a colloid perfusion fluid increases the recovered volume, but has no significant influence on the concentration of the sampled molecules at a perfusion rate of 2 μl/min[28]. Thus a colloid perfusion fluid would probably increase the recovered volume and more frequently allow triplicate analyses of each sample and larger volumes in each analysis.

The method as presented in this study is well suited for monitoring substantial changes in the composition of the epithelial lining fluid as seen with major physiological trauma such as sepsis and ischemia-reperfusion injury, and may even be used in the clinical setting. Combining the application of microdialysis in the bronchi with present knowledge of the microdialysis technique can provide better knowledge of the dynamics of the epithelial lining fluid in normal (pharmacokinetics) and pathological processes (altered permeability and measurement of markers of inflammation).

## Conclusion

Bronchial microdialysis is an applicable continuous monitor of the dynamic changes in the composition of the epithelial lining fluid. The absolute concentrations of the measured molecules can be estimated with acceptable precision using a correction by the arteriobronchial urea gradient.

Further research on bronchial microdialysis has to be done.

## Competing interests

The author(s) declare that they have no competing interests.

## Authors' contributions

SST, ES and PA have made major contributions to conception and design of the study. OL, SLS and SG have made major contributions to conception and design on laboratory methods and surgical preparations. All have contributed in the acquisition of data. SST, OL and ES have made major contributions in analysis and interpretation of the data. All have been involved in drafting the manuscript or revising it critically for important intellectual content. All have given final approval of the version to be published.
